# The randomized controlled trial Fast Track multilevel intervention for children with early‐emerging conduct problems breaks intergenerational transmission of violence across three generations

**DOI:** 10.1111/jcpp.70133

**Published:** 2026-02-11

**Authors:** Laura Gorla, W. Andrew Rothenberg, Jennifer Godwin, Conduct Problems Prevention Research Group, Karen L. Bierman, John D. Coie, D. Max Crowley, Kenneth A. Dodge, Mark T. Greenberg, Jennifer E. Lansford, John E. Lochman, Robert J. McMahon, Ellen E. Pinderhughes

**Affiliations:** ^1^ Center for Child and Family Policy Duke University Durham NC USA

**Keywords:** intergenerational, intimate partner violence, parent‐to‐child violence, intervention

## Abstract

**Background:**

Domestic violence mechanisms are frequently transmitted across generations, representing a global issue demanding particular attention. This study investigates the intergenerational transmission of intimate partner violence (IPV) and parent‐to‐child violence (PCV) and whether participating in a multilevel preventive intervention (Fast Track) breaks this transmission.

**Methods:**

In high‐risk elementary schools located in the United States, children considered at high risk for aggressive behavior based on teachers' and parents' screen scores were assigned to either a 10‐year intervention or a control group based on their school. The Fast Track trial was registered at clinicaltrials.gov (NCT01653535) and was focused on parenting practices and children's intrapersonal, interpersonal, and academic skills. From the original 891 children, 374 participants with children aged less than 18 years (*n* = 191 intervention group, *n* = 183 control group) reported at age 34 their experience with domestic violence and their children's psychological adjustment.

**Results:**

The intergenerational mediating pathway from high IPV in the first generation to high PCV in the second generation to greater total mental health difficulties in the third generation was statistically significant in the control group but not in the intervention group.

**Conclusions:**

IPV was intergenerationally transmitted by influencing PCV, with a negative effect on the third generation's mental health. Nevertheless, participation in the Fast Track intervention disrupted this cycle. These findings suggest the importance of policies to support preventive childhood interventions.

## Introduction

Globally, 27% of ever‐partnered women aged 15–49 years have experienced intimate partner violence in their lifetime (Sardinha, Maheu‐Giroux, Stöckl, Meyer, & García‐Moreno, 2022; World Health Organization, 2021). About 90 million children alive today have experienced sexual violence, and 1.6 billion children regularly face violent punishment and psychological aggression at home (UNICEF, 2024). In the United States alone, an estimated 10 million people are affected by domestic violence every year, and more than 25% of American children are exposed to family violence during their lives (Finkelhor, Turner, Ormrod, & Hamby, 2009; Huecker, King, Jordan, & Smock, 2025). Sadly, violence is often underreported and undocumented (Felson & Paré, 2005) and spreads across relationships in the family environment, being passed across generations (Kerr & Capaldi, 2019; Rothenberg, 2019) in cultures around the world (Lansford, Rothenberg, & Deater‐Deckard, 2022).

Domestic violence refers to physical, psychological, sexual, or emotional harm within the family, including intimate partner violence (IPV) and parent‐to‐child violence (PCV). Both direct (i.e., being personally victimized) and indirect (i.e., witnessing the victimization of a family member) exposure to domestic violence has detrimental effects on children's development (Gilbert, Widom, Browne, Fergusson, & Webb, 2009). Individuals who experienced or witnessed domestic violence are more likely to obtain lower educational achievements (Boden, Horwood, & Fergusson, 2007), engage in criminal actions (Lansford et al., 2021), and have internalizing and externalizing problems (Jaffee, Moffitt, Caspi, Taylor, & Arseneault, 2002; Tan & Mao, 2023). Experiencing domestic violence and being exposed to harmful and violent parenting practices also increase the risk of perpetrating violence in later relationships (Riedl et al., 2019; Salo, Appleton, & Tracy, 2022), being romantically involved with violent partners (Li, Zhao, & Yu, 2019), and using violent practices with one's own children (Lotto, Altafim, & Linhares, 2023; Savage, Tarabulsy, Pearson, Collin‐Vézina, & Gagné, 2019). Domestic violence is frequently transmitted across generations (Belsky, Conger, & Capaldi, 2009; Fitzgerald, London‐Johnson, & Gallus, 2020; Kerr & Capaldi, 2019; Rothenberg, 2019). The concept that individuals who have experienced domestic violence as victims may later become violent partners and parents has been explained as the intergenerational transmission of violence (Madigan et al., 2019), with violence mechanisms and effects stemming from grandparents to parents (first and second generation, G1 and G2, respectively) and then from parents to children (third generation, G3).

Given the adverse effects of domestic violence on development both in current and future generations, identifying protective factors against its concurrent and longitudinal effects is crucial. Participating in interventions to prevent violence and increase positive familial relationships can provide that protection (Pasalich, Fleming, Spieker, Lohr, & Oxford, 2019). Programs focusing on adopting positive parenting behaviors, targeting high‐risk samples (such as individuals showing emerging and escalating conduct problems at an early age) to prevent aggressive behaviors, or disrupting the pathways from problems in childhood to psychopathology in adulthood can break the intergenerational transmission of violence. Nevertheless, there is a paucity of longitudinal, multilevel, and multicomponent studies exploring how interventions have long‐term and enduring effects on different generations (Livings, Hsiao, & Withers, 2023). We fill this gap by testing whether participating in a multilevel preventive intervention (the Fast Track program, Conduct Problems Prevention Research Group [CPPRG]) breaks intergenerational transmission of intimate partner violence and parent‐to‐child violence across three generations.

The Fast Track intervention, launched in the early 1990s, was grounded in ecological and developmental theories that emphasize that chronic violence typically arises when children are exposed to multiple and sequential adversities (such as living in a dangerous neighborhood, experiencing a harsh home environment, and lacking support at school). As these risk factors interact and amplify, they accumulate over time, ultimately creating a persistent adverse condition that may have long‐lasting effects (McMahon et al., 1999). Given the cumulative harmful effects of these factors, providing a multicomponent and comprehensive intervention, involving both families and schools, is vital for breaking the pathways from conduct problems in childhood to violence in adulthood (Bierman, 2002). Therefore, the Fast Track intervention aimed at preventing lifelong violence in 5‐year‐olds whose parents and teachers identified as exhibiting early aggression, disruptive behavior, and other conduct problems in kindergarten. Families and children were initially included in the program, not for a history of domestic violence, but for elevated and significant behavior problems in children screened by both teachers and parents. Although the Fast Track intervention did not specifically address IPV, it aimed to prevent lifelong violence by comprehensively addressing major risk factors across different developmental periods, targeting children's intrapersonal, interpersonal, and academic skills, and their parents' parenting skills and behaviors. It combined comprehensive behavior‐management training for parents, child social and cognitive skills training, academic tutoring, home visits, and a universal curriculum to reduce disruptive and chaotic classroom environments from grades 1 through 10.

Fast Track positively impacted several aspects of parents' and children's lives from the early years. Compared to the control group, children and parents receiving the intervention displayed fewer aggressive behavior problems and decreased the use of physical punishment during elementary school (Bierman et al., 2004; CPPRG, 2011; McMahon et al., 1999). Youths receiving the intervention were less involved in delinquent actions during adolescence (CPPRG, 2007, 2010), and the intervention significantly reduced the need for general and mental health services through age 18 (Jones et al., 2010). As adults, Fast Track participants showed fewer externalizing problems and crime convictions (Dodge et al., 2015; Godwin & CPPRG, 2020; McCabe et al., 2024; Sorensen et al., 2016) and were less likely to use corporal punishment with their own children (Sorensen et al., 2016). Although all these results demonstrate the protective effects of the Fast Track intervention against violent behaviors, the current study is the first one to include three generations, therefore evaluating Fast Track's effects on domestic violence over a broader time span.

### The current study

The current study examines the intergenerational transmission of intimate partner violence and parent‐to‐child violence and whether participating in the Fast Track intervention disrupts these paths. We aimed to understand how past experiences of violence exposure within the G1–G2 family influence individuals from G2's violence in adulthood, both as a partner and parent, and affect children from G3's development. We also tested whether the Fast Track intervention acted as a protective factor for individuals from G2 exposed to violence during childhood, potentially disrupting the intergenerational transmission of violence. First, we hypothesized that parents from G2 with experiences of violence in their G1 family would be at higher risk of being violent with partners and children and would have children from G3 with lower psychological adjustment. Second, we hypothesized that parents from G2 who participated in the Fast Track intervention would report lower levels of violence toward their partners and children compared to those in the control group. Third, we expected that for individuals who participated in the Fast Track intervention, the negative effects of childhood violence would be disrupted across generations, thereby preserving the well‐being of the next generation.

## Methods

### Participants and recruitment

**Figure 1:**
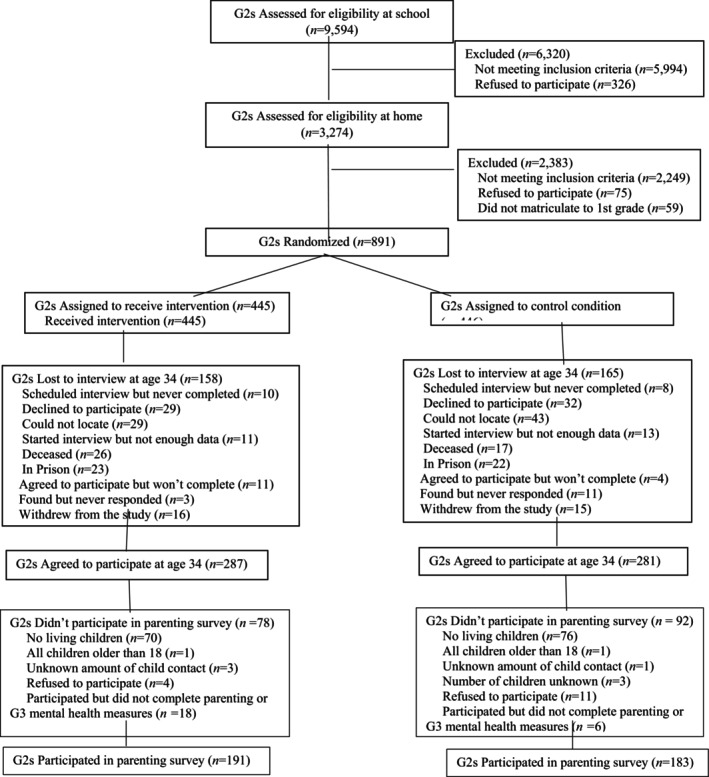
CONSORT flow chart (adapted from CPPRG, 2020)

High‐risk elementary schools (*n* = 53) were selected for Fast Track participation based on neighborhood crime and poverty rates in Durham, NC; Nashville, TN; rural Pennsylvania; and Seattle, WA. Within these schools, teachers screened 9,594 kindergarteners in three consecutive cohorts (1991–1993) for aggressive behavior. Those children scoring in the top 40% within each cohort and site were selected for the next screening of home behavior problems by parents. Teachers' and parents' screening scores were standardized and combined into a severity‐of‐risk screen score. Recruitment began with the highest‐scoring child and continued until designated sample sizes were reached within sites, cohorts, and groups. Within each site, the schools were divided into one to three paired sets of schools, and one set in each pair was randomly assigned to intervention and control conditions. 91% of recruited families agreed to participate, yielding a sample of 891 children from G2 (intervention group, *N* = 445; control group, *N* = 446; see Figure 1 CONSORT Diagram and CPPRG, 2020 for further details). At the selection, the participants' mean age was 6.58 years (*SD* = 0.48). Race varied (Black, 51%; white, 47%; other, 2%), and 69% were boys.

### Trial design

Written consent from parents and oral assent from children were obtained. Starting from 1992, parents from G1 (*M*_G1Age_ at first report = 30.70 years (*SD* = 6.44), Black, 48.51%; white, 49.75%, other, 1.74%, 97.51% women) reported on their parenting practices and intimate partner violence behaviors when children from G2 were ages 6, 13, and 14. Parents from G1 were paid for completing interviews. In 2020–2021, when the original children from G2 were adults and age 34, 374 participants from G2 (92% of eligible G2s; *n* = 191 intervention group, *n* = 183 control group) were invited to complete a survey that assessed their relationship with their romantic partner (if they had one), and their parenting of their children from G3. They also provided information on the mental health of their children (i.e., children from G3) if they had at least one child <18 years old and lived 20% or more of the time with their child or reported seeing/communicating with their child or the child's custodial guardian > monthly (see Figure 1 for data on attrition), with parenting practices and linked mental health measures applied to a single randomly selected G3 child per family (*M*_G3age_ = 9.37 years, *SD* = 4.37, 52% male). Participants completed the survey via an email link, on the phone, or in‐person and were modestly compensated for participation.

### Procedures

#### Intervention procedures

Intervention components were delivered to the first two generations (i.e., G1 parents and G2 children) from 1st through 10th Grade, with first sessions beginning in October of Grade 1. During elementary school, all families in the intervention group were offered parent training with home visiting, academic tutoring, and social skills. In particular, 2‐h parent and child group interventions involving parents from G1 and children from G2 were conducted at the school building on Saturdays or weekday evenings and included simultaneous children's social skill training and parent–child behavior‐management training (60 min), parent–child sharing time (30 min), and academic tutoring (30 min; Bierman et al., 2017). These group interventions were held weekly during first grade, biweekly during second grade, monthly from third to fifth grade, and four times per year during sixth grade. Furthermore, a teacher‐implemented, universal social–emotional learning curriculum (Kusché & Greenberg, 2020) was provided to children from G2 for an average of 2 to 3 lessons per week at all sites through Grade 5 except for Durham, NC (where school mergers after grade 1 prohibited further implementation). During grades 5–6, children from G2 received a middle school transition program and four parent‐youth groups on topics of adolescent development (alcohol/tobacco/drugs/decision‐making). In grades 7–8, eight youth forums were held to teach children from G2 about vocational opportunities, life skills, and summer employment. In grades 7–10, individualized interventions to parents from G1 and children from G2 targeted parent monitoring, peer affiliations, academic achievement, and social cognition (CPPRG, 2020). The families in the control group were followed over time, completed all questionnaires, but did not receive any intervention (therefore, they were free to seek other services if needed). All procedures were approved by the Institutional Review Boards of participating universities. Instructions for requesting and using Fast Track data are available at https://fasttrackproject.org/requesting‐and‐using‐data‐2/.

### Intervention integrity

Parent and child participation in programming and implementation fidelity were high. During grade 1, 96% of parents and 98% of children attended at least one group session. Of these, 79% of parents and 90% of children participated in at least 50% of scheduled sessions. Participation slightly declined across years (mainly due to residential moves) and, from grades 7 to 10, the intervention continued with at least 80% of all children. High intervention fidelity was ensured by manualization, regular cross‐site training, and weekly clinical supervision (Dodge et al., 2015).

### Measures

#### Primary outcome: children with G3's mental health problems

When individuals from G2 were age 34, children from G3's mental health was measured by parents from G2 report with the Strengths and Difficulties Questionnaire (SDQ; Goodman, Lamping, & Ploubidis, 2010; Goodman, 2001), a 25‐item normed measure designed to capture children's mental health difficulties. Parents were asked to indicate whether it was 0 = *not true*, 1 = *somewhat true*, or 2 = *certainly true* that their child demonstrated a behavior over the last 6 months. The 25 items were then evenly divided among 5 subscales. In the current study, we only used the 20 items related to behavioral problems and did not investigate the prosocial subscale. These subscales include conduct problems (α = .68), emotional problems (α = .70), hyperactivity/inattention (α = .80), and peer problems (α = .54). Items on each subscale were summed to create subscale scores. We also examined the total difficulties score, which sums scores of items on all subscales (α = .85).

#### Predictors: individuals from G1's intimate partner violence and parent to child violence

Individuals from G1's IPV and PCV, as well as individuals from G2's PCV, were each derived from the Conflict Tactics Scale (Straus, 1979). The Conflict Tactics Scale asks respondents how frequently they use specific behaviors with their child or romantic partner on a scale from 0 = *never* to 6 = *almost every day*. This study derived measures of individuals from G1's IPV and PCV, and individuals from G2's PCV, from two subscales of the Conflict Tactics Scale that can be calculated from interactions between parents and interactions between parents and children. These subscales are (1) the 6‐item verbal aggression subscale (e.g., “yelled, insulted, or swore,” “threatened to throw something”) and (2) the 4‐item physical aggression subscale (e.g., “pushed, grabbed, or shoved,” “hit or tried to hit”). Means across items are taken to calculate subscale scores.

Individuals from G1's IPV was measured by parents from G1's reports of their physical and verbal aggression directed toward their partner and, where available, partner from G1's reports of their physical and verbal aggression directed toward the parent from G1 when these measures were reported at child from G2 ages 6, 13, and 14. A composite measure of IPV was created by calculating a mean score across all parents and partners from G1 reports of physical and verbal aggression at all three of these time points. Creation of this composite score was justified by very high internal consistencies and strong positive correlations between parent and partner from G1 physical aggression across all 3 years (α = .75, *r* = .58, *p* < .01), parent and partner from G1 verbal aggression across all 3 years (α = .77, *r* = .74, *p* < .01), and between a composite measure of parent and partner from G1 verbal aggression and physical aggression (α = .80, *r* = .73, *p* < .01).

Individuals from G1's PCV were similarly measured by both parent and partner from G1's reports of physical and verbal aggression directed toward their child when these measures were reported at child from G2 ages 6–8 and 13–14. As with individuals from G1's IPV, a composite measure of violent parenting was created by calculating a mean score of all available reports across these time points. As with the individuals from G1's IPV composite measure, this measure demonstrated high internal consistency (α = .77) and positive correlations between parent and partner from G1's aggressive behavior (*r* = .35, *p* < .01).

#### Mediators: individuals from G2's intimate partner violence and parent to child violence

In the age 34 survey, individuals from G2's PCV was assessed using the same version of the Conflict Tactics Scale described for the individuals from G1 (see below), whereas a modified version of the Conflict Tactics Scale was used for physical and verbal aggression directed toward one's partner. Individuals from G2's IPV was measured by summing the frequency with which G2s reported engaging in any of 5 acts of violence (e.g., “you yelled or screamed at romantic partner”) or experiencing any of 15 acts of violence at the hand of their partner (e.g., “your romantic partner punched, hit, kicked, or slapped you”) in the past 12 months on a 0 = *never*, 1 = *once*, 2 = *2 times*, 3 = *3+ times* scale. This composite measure demonstrated high internal consistency (α = .79). The age 34 G2 survey did not have partner from G2's reports and was taken at only one time point. The individuals from G2's PCV composite measure was a mean of parents from G2's reports of their physical and verbal aggression toward their child. This measure also demonstrated high internal consistency (α = .76).

### Analytic plan

**Figure 2:**
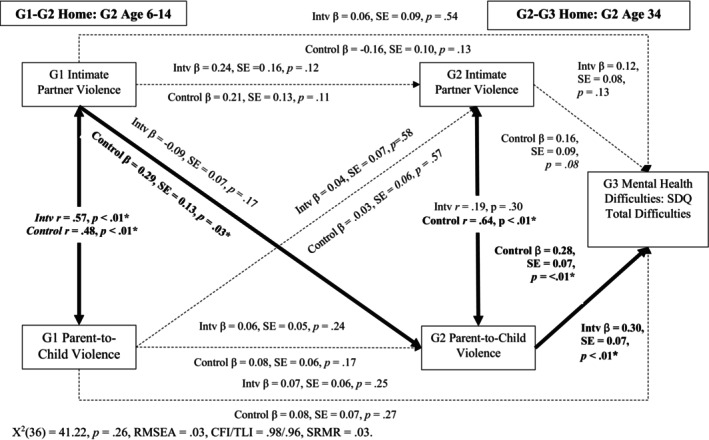
Path analysis: Intergenerational Transmission of Violence and Its Impact on G3 SDQ Total Difficulties Scores. Bolded path and * indicate *p* < .05. Dashed lines indicate path statistically nonsignificant in both groups. Solid lines indicate path statistically significant in at least one group. Intv: Fast Track Intervention Group. Control = Fast Track Control Group. SDQ = Strengths and Difficulties Questionnaire. G1 = Generation 1, G2 = Generation 2, G3 = Generation 3. Nine associations with covariates emerged in zero‐order correlations that were therefore controlled in the present model, but not pictured due to space constraints. They were the associations between (1) G2 IPV and G1 Parent Depression when G2s were 6. (2) G2 IPV and G2 Hostile Attribution Bias when they were 6. (3) G2 IPV and G1‐G2 Household SES. (4) G2 IPV and G2 participation in the 1993 cohort of G2s. (5) G2 IPV and G2 Total # correct on the Child Emotional Recognition Sum Score at Age 6. (6) G2 PCV and G2 participation in the Seattle, Washington cohort. (7) G3 SDQ Total Difficulties Scores and participation in the 1993 cohort of G2s. (8) G3 SDQ Total Difficulties Scores and the school the G2 went to at age 6. (9) G3 SDQ Total Difficulties Scores and G2 participation in the Durham, North Carolina cohort. Contact the second author if interested in these covariate associations

**Figure 3:**
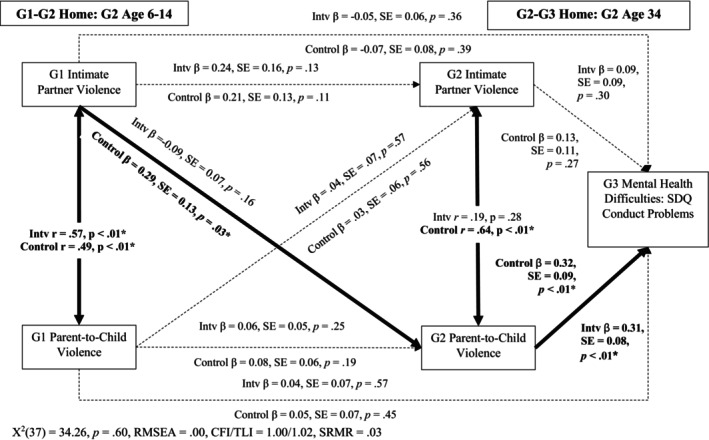
Path analysis: Intergenerational Transmission of Violence and Its Impact on G3 SDQ Conduct Problems. Bolded path and * indicate *p* < .05. Dashed lines indicate path statistically nonsignificant in both groups. Solid lines indicate path statistically significant in at least one group. Intv: Fast Track Intervention Group. Control = Fast Track Control Group. SDQ, Strengths and Difficulties Questionnaire. G1 = Generation 1, G2 = Generation 2, G3 = Generation 3. Nine associations with covariates emerged in zero‐order correlations that were therefore controlled for in the present model, but not pictured due to space constraints. They were the associations between (1) G2 IPV and G1 Parent Depression when G2s were 6. (2) G2 IPV and G2 Hostile Attribution Bias when they were 6. (3) G2 IPV and G1‐G2 Household SES. (4) G2 IPV and G2 participation in the 1993 cohort of G2s. (5) G2 IPV and G2 Total # correct on the Child Emotional Recognition Sum Score at Age 6. (6) G2 PCV and G2 participation in the Seattle, Washington cohort. (7) G3 SDQ Conduct Problems Scores and G2 Total # correct on the Child Emotional Recognition Sum Score at Age 6. (8) G3 SDQ Conduct Problems Scores and the school the G2 went to at age 6. (9) G3 SDQ Conduct Problems Scores and G2 participation in the Durham, North Carolina cohort. Contact the second author if interested in these covariate associations

**Figure 4:**
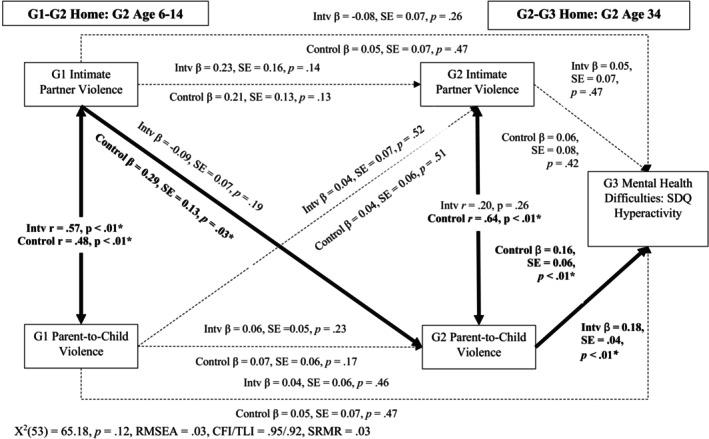
Path analysis: Intergenerational Transmission of Violence and Its Impact on G3 SDQ Hyperactivity. Bolded path and * indicate *p* < .05. Dashed lines indicate path statistically nonsignificant in both groups. Solid lines indicate path statistically significant in at least one group. Intv: Fast Track Intervention Group. Control = Fast Track Control Group. SDQ = Strengths and Difficulties Questionnaire. G1 = Generation 1, G2 = Generation 2, G3 = Generation 3. Thirteen associations with covariates emerged in zero‐order correlations that were therefore controlled for in the present model, but not pictured due to space constraints. They were the associations between (1) G2 IPV and G1 Parent Depression when G2s were 6. (2) G2 IPV and G2 Hostile Attribution Bias when they were 6. (3) G2 IPV and G1‐G2 Household SES. (4) G2 IPV and G2 participation in the 1993 cohort of G2s. (5) G2 IPV and G2 Total # correct on the Child Emotional Recognition Sum Score at Age 6. (6) G2 PCV and G2 participation in the Seattle, Washington cohort. (7) G3 SDQ Hyperactivity Scores and G3 age at assessment. (8) G3 SDQ Hyperactivity Scores and G2 race. (9) G3 SDQ Hyperactivity Scores and G2 participation in the 1993 cohort of G2s. (10) G3 SDQ Hyperactivity Scores and G2 sex. (11) G3 SDQ Hyperactivity Scores and the school G2s attended at age 6. (12) G3 SDQ Hyperactivity Scores and G2 participation in the Durham, North Carolina cohort. (13) G3 SDQ Hyperactivity Scores and G2 participation in the rural Pennsylvania cohort. Contact the second author if interested in these covariate associations

**Figure 5:**
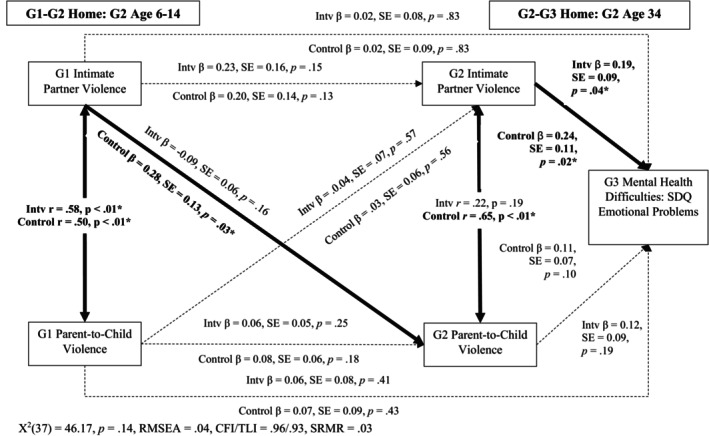
Path analysis: Intergenerational Transmission of Violence and Its Impact on G3 SDQ Emotional Problems. Bolded path and * indicate *p* < .05. Dashed lines indicate path statistically nonsignificant in both groups. Solid lines indicate path statistically significant in at least one group. Importantly, though both the intervention and control group parameter estimates for the effect of G2 Intimate Partner Violence on G3 Mental Health Difficulties: SDQ Emotional Problems were statistically significant at the *p* < .05 level, neither were significant at the Bonferroni‐corrected *p* < .0125 level. Intv: Fast Track Intervention Group. Control = Fast Track Control Group. SDQ, Strengths and Difficulties Questionnaire. G1 = Generation 1, G2 = Generation 2, G3 = Generation 3. Nine associations with covariates emerged in zero‐order correlations that were therefore controlled for in the present model, but not pictured due to space constraints. They were the associations between (1) G2 IPV and G1 Parent Depression when G2s were 6. (2) G2 IPV and G2 Hostile Attribution Bias when they were 6. (3) G2 IPV and G1‐G2 Household SES. (4) G2 IPV and G2 participation in the 1993 cohort of G2s. (5) G2 IPV and G2 Total # correct on the Child Emotional Recognition Sum Score at Age 6. (6) G2 PCV and G2 participation in the Seattle, Washington cohort. (7) G3 SDQ Emotional Problems Scores and G3 sex. (8) G3 SDQ Emotional Problems Scores and G2 Hostile Attribution Biase at age 6. (9) G3 SDQ Emotional Problems Scores and G2 sex. Contact the second author if interested in these covariate associations

**Figure 6:**
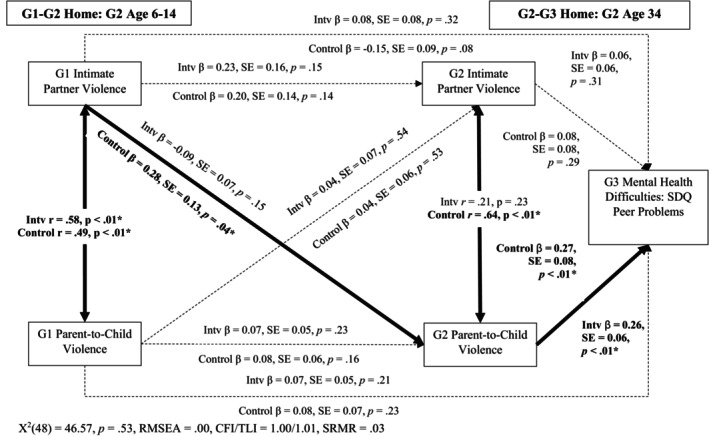
Path analysis: Intergenerational Transmission of Violence and Its Impact on G3 SDQ Peer Problems. Bolded path and * indicate *p* < .05. Dashed lines indicate path statistically nonsignificant in both groups. Solid lines indicate path statistically significant in at least one group. Intv: Fast Track Intervention Group. Control = Fast Track Control Group. SDQ = Strengths and Difficulties Questionnaire. G1 = Generation 1, G2 = Generation 2, G3 = Generation 3. Twelve associations with covariates emerged in zero‐order correlations that were therefore controlled for in the present model, but not pictured due to space constraints. They were the associations between (1) G2 IPV and G1 Parent Depression when G2s were 6. (2) G2 IPV and G2 Hostile Attribution Bias when they were 6. (3) G2 IPV and G1‐G2 Household SES. (4) G2 IPV and G2 participation in the 1993 cohort of G2s. (5) G2 IPV and G2 Total # correct on the Child Emotional Recognition Sum Score at Age 6. (6) G2 PCV and G2 participation in the Seattle, Washington cohort. (7) G3 SDQ Peer Problems Scores and G3 age at assessment. (8) G3 SDQ Peer Problems Scores and G2 participation in the 1991 cohort of G2s. (9) G3 SDQ Peer Problems Scores and G2 Total # correct on the Child Emotional Recognition Sum Score at Age 6. (10) G3 SDQ Peer Problems Scores and G2 sex. (11) G3 SDQ Peer Problems Scores and the school G2s attended at age 6. (12) G3 SDQ Peer Problems Scores and the mean % of G2 Child Competent Social Problem Solving Score at age 6. Contact the second author if interested in these covariate associations

Path analyses in Mplus Version 8.2 (Muthén & Muthén, 1998–2017) were run to investigate study questions. Specifically, path models in which individuals from G1's Intimate Partner Violence and Parent‐to‐Child violence each predicted individuals from G2's Intimate Partner Violence and Parent‐to‐Child violence, and children from G3's Mental Health Difficulties, and in which both individuals from G2's Intimate Partner Violence and Parent‐to‐Child violence also predicted children from G3's Mental Difficulties, were run. These paths were analyzed in multiple group models to test whether they differed by intervention status. First, eight paths were constrained to be equal across intervention and control groups. These paths included (1) individuals from G1's IPV predicting individuals from G2's IPV, (2) individuals from G1's PCV predicting individuals from G2's PCV, (3) individuals from G1's IPV predicting individuals from G2's PCV, (4) individuals from G1's PCV predicting individuals from G2's IPV, and the following paths predicting children from G3's mental health difficulties: (5) individuals from G1's IPV, (6) individuals from G1's PCV, (7) individuals from G2's IPV, and (8) individuals from G2's PCV. Then, one by one, these paths were freed to vary across groups, and the difference in fit was compared with a 1‐degree‐of‐freedom chi‐square test. If the chi‐square test revealed the model fit better when the path was allowed to freely vary across groups, the path remained unconstrained. In this way, we could identify exactly which intergenerational pathways differed across the Fast Track intervention and control groups. All 29 of the pre‐intervention and demographic covariates listed in Table S1, as well as children from G3's age and sex, were examined as potential control variables (see www.fasttrackproject.org; Bierman et al., 2004; CPPRG, 2007). Controlling for all 29 covariates when examining G1, G2, and G3‐related constructs simultaneously led to model under‐identification, so only covariates found to be significantly associated with each of the G1, G2, and G3‐related constructs in zero‐order correlations were controlled in the main analyses (see notes in Figures 2, 3, 4, 5, 6). Intervention status was coded as 0 = *control group member*, 1 = *intervention group member*.

In each path model, contemporaneous measures were correlated with one another (e.g., G1 measures were correlated with one another), and any of the 29 covariates that emerged as significant correlates in zero‐order correlations of measures referring to first, second and third generation were included in the model. Robust maximum likelihood estimation procedures were used to estimate path models to account for skewness or kurtosis in study variables and to ensure intent‐to‐treat analytic frameworks were followed. Notably, 35% of individuals from G2 (*n* = 141) were missing data on at least one of the G1, G2, or G3 measures. However, individuals from G2 with versus without missing data did not differ in their membership in the intervention versus control group or in any of the IPV, PCV, or children from G3 mental health difficulties variables. All 403 individuals from G2 who had data on G1, G2, or G3 constructs (even those with partially missing data) were included in all analyses.

Five path models were run that were identical in structure with each exploring one of the 4 SDQ subscales and the total score that captured children from G3's mental health difficulties. In each of the five path models, Mplus's MODEL INDIRECT command was used to capture the mediational pathways from individuals from G1's IPV and individuals from G1's PCV to children from G3's mental health difficulties through individuals from G2's IPV and PCV (Muthén & Muthén, 1998–2017). The MODEL INDIRECT command calculates the mediational effect using the product of path coefficients method and estimates standard errors using the delta method (Muthén & Muthén, 1998–2017).

## Results

### Baseline and intergenerational group differences and attrition rates

The parents from G2 subsample and the initial sample from G2, and the parents from G2 intervention and control samples only differed on a combined 8 of 58 tests of pre‐treatment and demographic differences between groups (see Table S1). Specifically, men from G2 and individuals from G2 who had higher externalizing scores or social competence scores at age 6 were less likely to participate at age 34. Individuals from G2 in the age 34 intervention group had lower age 6 friendship satisfaction scores, but higher age 6 social competence scores and lower externalizing behavior risk scores, compared to those in the control group. Of these differences, only the fact that individuals from G2 being male was significantly associated with any individuals from G2's IPV or PCV, or any children from G3's mental health problems. This variable was controlled in all applicable analyses.

### Intergenerational transmission of violence and its impact on G3 behavior problems

Table S2 reports descriptive statistics and zero‐order correlations for the main study variables. Final omnibus model fit statistics are depicted at the bottom of each figure and indicate that each model fits the data excellently (e.g., CFI/TLI ≥ .95, RMSEA ≤ .06, SRMR ≤ .08; Hu & Bentler, 1999). The results sections summarize the main findings; exact parameter estimates are reported in Figures 2, 3, 4, 5, 6; see Appendix S1 for a complete description of results from all models. All reported parameter estimates are standardized and all statistically significant findings survived Bonferroni correction (Bonferroni, 1936) except for two (noted below).

After examining chi‐squared difference tests, the models predicting (1) Children from G3's SDQ Total Difficulties (Figure 2), (2) Conduct Problems (Figure 3), and (3) Hyperactivity (Figure 4) all fit best when the path from individuals from G1's IPV predicting individuals from G2's PCV was freed to vary across the Fast Track intervention and control groups (for further detail, refer to Appendix S1). This indicates that the magnitude of the pathway from individuals from G1's IPV to individuals from G2's PCV varied across the FT treatment and control groups. The final models are depicted in Figures 2, 3, 4.

All three models support our first hypothesis that individuals from G2 with experiences of violence in their G1–G2 family would engage in greater violence in their G2–G3 adult families, leading to lower psychological adjustment for their children. Specifically, for individuals from G2 in the control group, if parents from G1 reported engaging in higher IPV when individuals from G2 were 6–14 years old, then those individuals from G2 engaged in higher levels of PCV at age 34, which were associated with greater children from G3's total mental health difficulties, conduct problems, and hyperactivity.

These models also supported our second hypothesis that the Fast Track intervention protects against this intergenerational transmission of violence and its consequences. Specifically, individuals from G1's higher IPV predicted individuals from G2's higher PCV in the control group but not in the Fast Track intervention group in all three models (Figures 2, 3, 4; Table 1). Consequently, the intergenerational mediating pathways from individuals from G1's high IPV to individuals from G2's high PCV to children from G3's greater mental health difficulties were statistically significant in the control group (SDQ Total Difficulties Indirect Effect = 0.93, *SE* = 0.45, 95% CI: 0.03–1.83, *p* = .04; SDQ Conduct Problems Indirect Effect = 0.27, *SE* = 0.13, 95% CI: 0.01–0.53, *p* = .03; SDQ Hyperactivity Indirect Effect = 0.24, *SE* = 0.12, 95% CI: 0.005–0.48, *p* = .05), but not in the intervention group (SDQ Total Difficulties Indirect Effect = −0.35, *SE* = 0.30, 95% CI: −0.95 to 0.25, *p* = .25; SDQ Conduct Problems Indirect Effect = −0.11, *SE* = 0.09, 95% CI: −0.29 to 0.07, *p* = .23; SDQ Hyperactivity Indirect Effect = −0.09, *SE* = 0.08, 95% CI: −0.25 to 0.07, *p* = .25). All three of these effects represented full mediation; the direct effect of individuals from G1's IPV on children from G3's SDQ Total Difficulties, Conduct Problems, and Hyperactivity was nonsignificant after taking this mediating pathway into account.

**Table 1:** Total, direct, and indirect effects for each G1 intimate partner violence → G2 parent–child violence → G3 mental health difficulty pathway

Mediating pathway	Fast Track Control Group						Fast Track Intervention Group					
	Total effect (*SE*)	*p*	Direct effect (*SE*)	*p*	Indirect effect (*SE*)	*p*	Total effect (*SE*)	*p*	Direct Effect (*SE*)	*p*	Indirect Effect (*SE*)	*p*
G1 IPV → G2 PCV → G3 SDQ total difficulties	−0.45 (0.91)	.63	−1.76 (1.10)	.11	**0.93 (0.45)**	.04*	0.78 (1.32)	.56	0.75 (1.20)	.53	−0.35 (0.30)	.25
G1 IPV → G2 PCV → G3 SDQ conduct problems	0.16 (0.22)	.48	−0.19 (0.22)	.37	**0.27 (0.13)**	.03*	−0.22 (0.22)	.32	−0.19 (0.22)	.37	−0.11 (0.09)	.33
G1 IPV → G2 PCV → G3 SDQ hyperactivity	−0.13 (0.37)	.73	−0.43 (0.39)	.26	**0.24 (0.12)**	.05*	−0.45 (0.37)	.23	−0.43 (0.39)	.26	−0.09 (0.08)	.25
G1 IPV → G2 PCV → G3 SDQ emotional problems	0.34 (0.29)	.24	0.06 (0.29)	.83	0.11 (0.07)	.11	0.19 (0.31)	.54	0.06 (0.29)	.83	−0.04 (0.05)	.40
G1 IPV → G2 PCV → G3 SDQ emotional problems	−0.19 (0.26)	.46	−0.49 (0.27)	.07	0.24 (0.13)	.07	0.39 (0.24)	.11	0.30 (0.24)	.21	−0.10 (0.08)	.21

* and Bold indicates statistically significant at *p* < .05. SDQ, Strengths and Difficulties Questionnaire. For simplicity, individuals from G1 and G2's Intimate Partner Violence and Parent‐to‐Child Violence were labeled as “G1 IPV”, “G1 PCV”, “G2 IPV”, and “G2 PCV”. Similarly, children from G3's SDQ variables were called “G3 SDQ”.

With regard to other significant findings, across all three models, in both the control and intervention groups, individuals from G2's higher levels of PCV were associated with children from G3's greater total mental health difficulties (Figure 2), conduct problems (Figure 3), and hyperactivity (Figure 4). Additionally, in both the intervention and control groups in all three models, individuals from G1's IPV and PCV were highly correlated. However, in all three models, in the control group, individuals from G2's IPV and PCV were highly correlated, whereas they were not significantly correlated in the intervention group. No other pathways in these models were statistically significant.

### Intergenerational transmission of violence and its impact on G3 emotional problems

Similar to Models 1–3, the children from G3's SDQ Emotional Problems model (Figure 5) fit best when the path from individuals from G1's IPV predicting individuals from G2's PCV was freed to vary across groups, indicating that this path varied significantly in magnitude across the FT treatment and control groups (Figure 4).

However, unlike in Models 1–3, hypotheses 1 and 2 were only partially supported. Specifically, once again, individuals from G1's higher IPV predicted individuals from G2's higher PCV in the control group but not the Fast Track intervention group. However, in both groups, individuals from G2's PCV were not significantly associated with children from G3's SDQ emotional problems. Instead, individuals from G2's higher IPV were significantly associated with children from G3's higher SDQ emotional problems in both groups, though the statistical significance of these two positive associations did not remain after Bonferroni correction (as *ps* = .02–.04, short of Bonferroni‐corrected *p* of .0125). Therefore, these results partially supported hypothesis 1 because individuals from G1's violent behavior (in the form of IPV) did predict individuals from G2's violent behavior (in the form of PCV) in the control group, and they partially supported hypothesis 2 because Fast Track participation protected against this link in the treatment group. However, both hypotheses were not fully supported because individuals from G2's violent parenting were not associated with children from G3's emotional problems, and consequently the mediating path from individuals from G1's IPV to individuals from G2's PCV to children from G3's emotional problems was not statistically significant in either the control (Table 1; Indirect Effect = 0.11, *SE* = 0.07, 95% CI: −0.25 to 0.03, *p* = .11) or intervention (Table 1; Indirect Effect = −0.04, *SE* = 0.05, 95% CI: −0.14 to 0.06, *p* = .40) group.

### Intergenerational transmission of violence and its impact on G3 peer problems

The children from G3's SDQ Peer Problems model (Figure 6) results were similar to the models for children from G3's total difficulties, conduct problems, and hyperactivity models, as it fit best when the path from individuals from G1's IPV predicting individuals from G2's PCV was freed to vary across groups indicating a significant difference in magnitude of this path across groups. The significance of the individual paths in this model replicated the models for children from G3's total difficulties, conduct problems, and hyperactivity. Specifically, for individuals from G2 in the control group, if parents from G1 reported engaging in higher IPV when individuals from G2 were 6–14 years old, then those individuals from G2 engaged in higher levels of PCV at age 34, which were associated with children from G3's greater peer problems. However, the Fast Track intervention protected against this intergenerational transmission of violence because the link between individuals from G1's IPV and individuals from G2's PCV was only significant in the control group, not the intervention group.

The model for children from G3's peer problems differed from the children from G3's total difficulties, conduct problems, and hyperactivity models when the entire intergenerational mediating pathway is examined. The mediating pathway from individuals from G1's IPV to individuals from G2's PCV to children from G3's peer problems is not statistically significant in either the control (Table 1; Indirect Effect = 0.24, *SE* = 0.13, 95% CI: −0.02 to 0.50, *p* = .07) or intervention (Table 1; Indirect Effect = −0.10, *SE* = 0.08, 95% CI: −0.26 to 0.06, *p* = .21) groups. Therefore, like the children from G3's emotional problems model, hypotheses 1 and 2 are only partially supported in the children from G3's peer problems model.

## Discussion

We examined the intergenerational transmission of IPV and PCV, their effects on the third generation, and the role of the Fast Track intervention as a potential disruptor of this intergenerational path. Our results showed that intimate partner violence was transmitted across generations by influencing parent‐to‐child violence. Indeed, parents from G2 who witnessed high IPV between their parents (i.e., individuals from G1) during their childhood and early adolescence were likely to act more violently toward their children (i.e., children from G3). This intergenerational path has adverse effects on the next generation's well‐being, as children from G3 suffer more from psychological difficulties, showing higher levels of conduct problems and hyperactivity. However, participation in the Fast Track intervention disrupted this intergenerational transmission of violence.

Curiously, no intergenerational links were found between domain‐specific forms of violence, as individuals from G1's IPV were not associated with individuals from G2's IPV, nor were individuals from G1's PCV connected to individuals from G2's PCV. Although this direct intergenerational path was absent, significant correlations between IPV and PCV emerged in the first generation for both the intervention and control groups and in the second generation for only the control group. These findings suggest that, although IPV and PCV were concurrently related in the first and second generations, only cross‐construct associations between IPV and PCV were transmitted across generations, ultimately undermining children from G3's psychological adjustment. These results reinforce the understanding of domestic violence as a complex phenomenon, where disentangling direct and indirect exposure and distinguishing different forms of violence can be challenging (Riedl et al., 2019; Thi, Zimmerman, Pocock, Chan, & Ranganathan, 2022). Still, IPV and PCV associations were intergenerationally transmitted only in the control group, suggesting the role of the Fast Track intervention in reducing the intergenerational violence transmission pathway. Interestingly, Fast Track acted as a protective factor not only across generations but also within generations, with parents from G2 reporting no significant correlations between their violence toward their partners and their children.

Overall, these findings align with previous research highlighting that individuals with a personal history of domestic violence have a greater likelihood of perpetrating violent behaviors toward their partners and children, perpetuating a cycle of violence with adverse effects across generations (Belsky et al., 2009; Fitzgerald et al., 2020; Riedl et al., 2019; Rothenberg, 2019; Salo et al., 2022). Moreover, they align with past studies reporting the harmful effects of PCV on children's psychological adjustment (Jaffee et al., 2002; Oram et al., 2022; Vu, Jouriles, McDonald, & Rosenfield, 2016). To our knowledge, the current study is the first to combine prospective longitudinal data about IPV and PCV across three generations, also testing for intervention effects on intergenerational violence transmission. It reveals that the Fast Track intervention breaks the cycle of violence by eliminating the transmission of individuals from G1's IPV in the form of individuals from G2's PCV and reducing the association between individuals from G2's IPV and PCV. This ability may stem from the Fast Track intervention's aims to prevent lifelong violence by comprehensively addressing major risk factors across different developmental periods and targeting several dimensions, such as intrapersonal and interpersonal skills of children and parents. Specifically, Fast Track emphasized building interpersonal skills and emotional competencies in individuals from G2. These skills included the ability to form and maintain positive relationships with peers, effectively regulate and communicate personal emotions, and resolve conflicts constructively. Through friendship groups, parent groups, parent–child sharing, and home visiting, Fast Track's intervention components targeted children's prosocial skills, emotional expression and understanding, accurate awareness of others' intentions, anger control, as well as parents' warmth, support, and reduction of harsh and punitive discipline (CPPRG, 2020; McCabe et al., 2024). Such competences are widely recognized as fundamental for positive psychological adjustment across the life span, as they protect against the development of aggressive and violent behavioral patterns (Lansford et al., 2006; Lösel & Farrington, 2012; Siegel, 2013). Past work has demonstrated that Fast Track's impacts on individuals from G2's later psychopathology can be accounted for by improvements in their social and self‐regulation skills during childhood (Godwin et al., 2020; McCabe et al., 2024; Sorensen et al., 2016) that subsequently may prevent the use of individuals from G2's violent parenting behaviors in adulthood (Rothenberg et al., 2023, 2024). This protective role of Fast Track's multilevel intervention confirms previous research findings highlighting that psychological intervention targeting both parents and children may reduce domestic violence and increase family members' well‐being (Livings et al., 2023).

Interestingly, although the intervention disrupted the transmission of violence from individuals from G1's IPV to individuals from G2's PCV and then from individuals from G2's PCV to children's G3 outcomes, the pathway between individuals from G2's PCV and children from G3's mental health difficulties remained significant. Several factors might account for this finding. First, other key variables might play a role in this path and warrant further investigation. For example, it is possible that the developmental tasks children deal with in 2020–2021 might have different characteristics compared to the ones experienced by individuals from G2 in the 1990s. In the context of modern societal changes, such as shifting family values, earlier pubertal onset, and more fluid social relationships, parents' violence influences on children's mental health might be slightly different, therefore changing the intervention's long‐lasting effects. Furthermore, it is possible that children from G3 whose parents' violent parenting behaviors endure despite the intervention are more likely to suffer from psychological difficulties, especially if individuals from G2 showed stronger aggressive behaviors during childhood. These results suggest additional research on the topic, such as longitudinally following children from G3's development to gain deeper insights into intergenerational pathways. Finally, these results might point to the need for ongoing monitoring and targeted support for individuals with a family history of violence, suggesting that breaking the intergenerational cycle of violence requires constant and sustained interventions.

The violence‐related paths within and across generations change slightly if we consider G3's emotional and peer problems. Indeed, for both children from G3's emotional and peer problems, the mediating pathway from individuals from G1's IPV to individuals from G2's PCV to G3's outcomes was not statistically significant, and emotional problems were not directly associated with individuals from G2's PCV. These findings suggest a difference between internalizing and externalizing problems in G3 children, with externalizing problems (i.e., conduct problems and hyperactivity) more likely to emerge because of the intergenerational transmission of violence compared to internalizing problems (i.e., emotional and peer problems). Notably, the original G2 sample was composed of children identified as exhibiting early externalizing problems. Following Bandura's (1969) social learning theory, G3 children may be more likely to exhibit increased externalizing behaviors by modeling their parents' behaviors. Parent‐to‐child intergenerational transmission of psychopathological symptoms, such as internalizing and externalizing problems, has been reported in previous research (Goodman et al., 2011; Marceau, Yu, Knopik, Ganiban, & Neiderhiser, 2022). Considering the current G2 sample's characteristics, future research focused on the domain‐specific intergenerational transmission of externalizing symptoms may be interesting to further confirm current results.

The study also has limitations. First, although we measured G1's IPV using both partners' reports and across several years, we did not possess G2 partner reports and collected G2's IPV data only at one time point. Second, the SDQ peer problems subscale presented moderate internal consistency, potentially influencing results by attenuating potential intergenerational pathways predicting these peer problems. Third, G3's psychological adjustment was reported by parents and was collected only at one time point. Finally, although these findings highlight the effectiveness of the Fast Track intervention, it is important to consider two aspects. The first one is that large‐scale implementation may be challenging due to its long duration and intensive nature. Not all interventions might span 10 years, involve both families and schools, and address several major risk factors across different developmental periods, as the Fast Track intervention did, especially given that implementing such interventions might be highly costly for public health providers. However, the Fast Track intervention has been proven to be cost‐effective, and possible cost‐effectiveness assessments for future implementations have been proposed (Foster et al., 2006). The second aspect, often highlighted in the violence research field, is the possible social desirability bias in self‐reports of violence. Although several studies underscored the issue of social desirability in individuals' responses to the Conflict Tactics Scale (Archer, 1999), others pointed out that confidentiality and anonymity might encourage full disclosure (Hamby, 2014) and that social desirability distortion on violence report is minimal (Visschers, Jaspaert, & Vervaeke, 2015). In the current study, although potential bias in self‐reports of violence should be acknowledged, we used both self and partner reports to create a composite score of IPV and PCV when possible, finding high endorsement rates between parents and partners (i.e., 94% of parents from G1 and 90% of partners from G1 reported at least one IPV behavior and 66% of parents from G1 and 88% of partners from G1 reported at least one PCV behavior). We believe that these actions likely decreased possible social desirability effects.

Despite these limitations, this study makes a significant contribution by demonstrating that violence is not confined to a single generation but persists across multiple generations; however, it can be disrupted by a multilevel preventive intervention. These findings suggest the importance of programs and policies to support childhood interventions to reduce conduct problems, which can pay dividends to children at the time of the intervention as well as when they become adults and even into the next generation.

## Ethical considerations

Written consent from parents and oral assent from children were obtained. All procedures were approved by the Duke University Institutional Review Board on June 2, 2025 (protocol number 2018–0331).

## Trial registration

The Fast Track trial was registered at clinicaltrials.gov (identifier number: NCT01653535). First submitted 2012‐07‐16. Available at https://clinicaltrials.gov/study/NCT01653535?intr=Fast%20Track&id=NCT01653535&rank=1.

**Key points**

**What's known?**

- Domestic violence is frequently transmitted across generations, with preventive interventions potentially breaking this transmission.

**What's new?**

- Still, few longitudinal studies are available. This study examines domestic violence transmission and the role of Fast Track, a multilevel preventive intervention for children at high risk for aggressive behaviors.
- Intimate partner violence was transmitted across generations by influencing parent‐to‐child violence, with greater psychological difficulties for the next generation. Still, these intergenerational patterns were not significant in the intervention group.

**What's relevant?**

- Fast Track eliminates the intergenerational transmission of intimate partner violence and parent‐to‐child violence.

## Supporting information


**Table S1.** Pre‐intervention & demographic means for the G2 parent subsample, G2 non‐parent sample, G2 intervention parent subsample, and G2 control parent subsample by intervention status.
**Table S2.** Main study variables descriptive statistics and correlations.
**Appendix S1.** Supplemental results section.

## Data Availability

The data that support the findings of this study are available from the corresponding author upon reasonable request.

## Appendix 1

The members of the Conduct Problems Prevention Research Group (CPPRG) are, in alphabetical order, Karen L. Bierman (Pennsylvania State University), John D. Coie (Duke University), D. Max Crowley (Pennsylvania State University), Kenneth A. Dodge (Duke University), Mark T. Greenberg (Pennsylvania State University), Jennifer E. Lansford (Duke University), John E. Lochman (University of Alabama), Robert J. McMahon (Simon Fraser University and the B.C. Children's Hospital Research Institute), and Ellen E. Pinderhughes (Tufts University).
