# Supplementary material for: The randomized controlled trial Fast Track multilevel intervention for children with early‐emerging conduct problems breaks intergenerational transmission of violence across three generations
Source: J Child Psychol Psychiatry. 2026 Feb 11;67(8):1254–68. doi: 10.1111/jcpp.70133 (PMC13182863; doi:10.1111/jcpp.70133)
Supplement: Supplementary file 1 — Table S1. Pre‐intervention & demographic means for the G2 parent subsample, G2 non‐parent sample, G2 intervention parent subsample, and G2 control parent subsample by intervention status. Table S2. Main study variables descriptive statistics and correlations. Appendix S1. Supplemental results section. [file JCPP-67-1254-s001.docx]

**The Fast Track Multilevel Intervention for Children with Early-emerging Conduct Problems Breaks Intergenerational Transmission of Violence Across Three Generations**

**Supporting Information**

**Table S1.** *Pre-Intervention & Demographic Means for the G2 Parent Subsample, G2 Non-Parent Sample, G2 Intervention Parent Subsample, and G2 Control Parent Subsample by Intervention Status*

|  | Original Sample | | | | Age 34 Parent Sample | | | |
| --- | --- | --- | --- | --- | --- | --- | --- | --- |
| Variable | Participated in Age 34 Parent Sample  (*n* = 374) | | Did Not Participate in Age 34 Parent Sample  (*n* = 517) | | G2 Intervention Parents  (*n* = 191) | | G2 Control Parents  (*n* = 183) | |
|  | *M or %* | *SD* | *M or %* | *SD* | *M or %* | *SD* | *M or %* | *SD* |
| Pre-Intervention Variables Measured in G1-G2 Family Before Start of Fast Track Intervention in 1991-1993 | | | | | | | | |
| Depression Score | 16.42 | 9.91 | 16.25 | 10.31 | 16.54 | 9.92 | 16.30 | 9.91 |
| % Hostile Attributions | 68% | 26% | 66% | 25% | 68% | 27% | 67% | 26% |
| Aggressive Behavior Score | 21.90 | 7.99 | 22.68 | 8.33 | 22.21 | 8.22 | 21.57 | 7.75 |
| Appropriateness Score | 3.54 | 0.79 | 3.58 | 0.73 | 3.56 | 0.76 | 3.52 | 0.83 |
| Family Satisfaction | 2.17 | 0.73 | 2.13 | 0.72 | 2.12 | 0.78 | 2.20 | 0.68 |
| Friendship Satisfaction | 2.37 | 0.62 | 2.31 | 0.60 | **2.28^b^** | 0.67 | **2.46^b^** | 0.51 |
| Physical Punishment Mean Score | 0.22 | 0.22 | 0.22 | 0.24 | 0.20 | 0.21 | 0.24 | 0.23 |
| Kindergarten Stress Scale | 5.23 | 4.25 | 5.31 | 4.00 | 5.32 | 4.41 | 5.13 | 4.08 |
| Verbal Punishment Mean Score | 0.26 | 0.31 | 0.26 | 0.32 | 0.26 | 0.34 | 0.26 | 0.28 |
| Socioeconomic Status | 23.55 | 12.14 | 24.97 | 13.04 | 23.78 | 12.56 | 23.31 | 11.71 |
| Oppositional Aggressive Score | 0.25 | 0.17 | 0.27 | 0.18 | 0.25 | 0.16 | 0.25 | 0.17 |
| Warm, Harsh, and Appropriate Discipline Mean | 2.00 | 0.27 | 2.00 | 0.28 | 1.99 | 0.27 | 2.00 | 0.28 |
| Social Competence Total Score | **2.09^a^** | **0.60** | **2.01^a^** | 0.58 | **2.15^b^** | **0.58** | **2.03^b^** | **0.62** |
| Letter and Word Identification Score | 12.55 | 4.00 | 12.66 | 4.89 | 12.86 | 4.31 | 12.24 | 3.63 |
| Total Number Correct on Emotion Recognition Questionnaire | 10.67 | 2.72 | 10.74 | 2.87 | 10.60 | 2.71 | 10.74 | 2.74 |
| Warmth | 3.58 | 0.74 | 3.58 | 0.82 | 3.62 | 0.75 | 3.53 | 0.73 |
| Mean % of Competent Responses to Social Problem Solving Scale | 62% | 22% | 63% | 22% | 62% | 21% | 61% | 23% |
| Neighborhood Questionnaire Total Score | -0.04 | 0.61 | -0.04 | 0.59 | -0.08 | 0.62 | 0.00 | 0.59 |
| Average Standard Wechsler Intelligence Scale for Children Score | -0.11 | 0.74 | -0.05 | 0.82 | -0.10 | 0.74 | -0.12 | 0.73 |
| Externalizing Behavior Risk Standardized Score | **0.81^a^** | 0.63 | **0.94^a^** | 0.71 | **0.74^b^** | **0.62** | **0.87^b^** | **0.63** |
| Demographic Variables | | | | | | | | |
| % Black | 50.27% |  | 48.55% |  | 51.07% |  | 48.93% |  |
| % Member of Cohort 1 | 37.70% |  | 32.69% |  | 37.70% |  | 37.70% |  |
| % Member of Cohort 2 | 34.49% |  | 37.14% |  | 35.60% |  | 33.33% |  |
| % Member of Cohort 3 | 27.81% |  | 30.17% |  | 26.70% |  | 28.96% |  |
| % Male | **57.49%^a^** |  | **77.95%^a^** |  | 59.69% |  | 55.19% |  |
| % From Durham, NC Site | 26.47% |  | 23.21% |  | 26.18% |  | 26.78% |  |
| % From Nashville, TN Site | 24.60% |  | 26.69% |  | 25.13% |  | 24.04% |  |
| % From Penn State, PA Site | **29.95%^a^** |  | **21.86%^a^** |  | 27.23% |  | 32.79% |  |
| % From Seattle, WA Site | **18.98%^a^** |  | **28.24%^a^** |  | 21.47% |  | 16.39% |  |

^a^Data represent significant difference (*p* < .05) between individuals from G2 who participated in age 34 parent sample and those who did not participate in age 34 parent sample according to an independent samples t-test for continuous variables or chi-square tests for categorical variables.

^b^Data represent significant difference (*p* < .05) between G2 intervention parents and G2 control parents according to an independent samples t-test for continuous variables or chi-square tests for categorical variables.

**Table S2.** *Main Study Variables Descriptive Statistics and Correlations*

|  | *N* | *M* (SD) or % | Range | 1. | 2. | 3. | 4. | 5. | 6. | 7. | 8. | 9. | 10. |
| --- | --- | --- | --- | --- | --- | --- | --- | --- | --- | --- | --- | --- | --- |
| 1. G1 IPV | 318 | 0.52 (0.49) | 0-3.75 | 1.00 |  |  |  |  |  |  |  |  |  |
| 2. G1 PCV | 403 | 0.81 (0.43) | 0-3.51 | **.51*** | 1.00 |  |  |  |  |  |  |  |  |
| 3. G2 IPV | 359 | 0.40 (1.30) | 0-15 | **.34*** | **.14*** | 1.00 |  |  |  |  |  |  |  |
| 4. G2 PCV | 373 | 0.26 (0.52) | 0-6 | **.19*** | **.12*** | **.49*** | 1.00 |  |  |  |  |  |  |
| 5. SDQ Total Difficulties | 374 | 8.16 (5.94) | 0-30 | .06 | .09 | **.25*** | **.34*** | 1.00 |  |  |  |  |  |
| 6. SDQ Conduct Problems | 374 | 1.26 (1.61) | 0-8 | .09 | .07 | **.25*** | **.36*** | **.77*** | 1.00 |  |  |  |  |
| 7. SDQ Emotional Problems | 374 | 1.55 (1.81) | 0-10 | **.11*** | **.12*** | **.25*** | **.24*** | **.73*** | **.44*** | 1.00 |  |  |  |
| 8. SDQ Hyperactivity | 374 | 3.52 (2.66) | 0-10 | -.05 | .01 | **.11*** | **.19*** | **.81*** | **.51*** | **.42*** | 1.00 |  |  |
| 9. SDQ Peer Problems | 374 | 1.83 (1.75) | 0-8 | .09 | .11* | **.19*** | **.27*** | **.69*** | **.47*** | **.42*** | **.32*** | 1.00 |  |
| 10. In the Fast Track Intervention Group | 403 | 51.36% | N/A | .02 | .01 | .00 | .00 | .00 | .04 | -.06 | -.01 | .04 | 1.00 |

*Note.* **p* < .05. IPV = Intimate Partner Violence. PCV = Parent-to-Child Violence. SDQ = Strengths and Difficulties Questionnaire.

**Appendix S1. Supplemental Results Section**

**Model 1: Intergenerational Transmission of Violence and Its Impact on G3 SDQ Total Difficulties Scores (Figure 2)**

After examining chi-squared difference tests, the model fit best when two of the aforementioned eight paths were freed to vary across Fast Track intervention and control groups: (1) the path from G1 IPV predicting G2 PCV (*Χ^2^*(1) = 13.40, *p* < .01) and (2) the path from G1 IPV predicting G3 SDQ Total Difficulties scores (*Χ^2^*(1) = 3.85, *p* = .05). This final model is depicted in Figure 2.

This model supports our first hypothesis that parents with experiences of violence in their G1G2 family would engage in greater violence in their adult families, leading to lower psychological adjustment for their children. Specifically, for G2s in the control group, if G1 parents reported engaging in higher IPV when G2s were 6-14 years old, then those G2s engaged in higher levels of PCV at age 34, which were associated with greater G3 total mental health difficulties.

However, this model also supported our second hypothesis that the Fast Track intervention protects against this intergenerational transmission of violence and its consequences. Specifically, higher G1 IPV predicted higher G2 PCV in the control group but not in the Fast Track intervention group. Consequently, the intergenerational mediating pathway from high G1 IPV to high G2 PCV to greater G3 total mental health difficulties was statistically significant in the control group (Indirect Effect = 0.93, SE = 0.45, 95% CI: 0.03-1.83, *p* = .04), but not in the intervention group (Indirect Effect = -0.35, SE = 0.30, 95% CI: -0.95-0.25, *p* = .25).

With regards to other significant findings, in both the control and intervention groups, higher levels of G2 PCV were associated with greater G3 total mental health difficulties. Additionally, in both the intervention and control groups, G1 IPV and G1 PCV were highly correlated. However, in the control group, G2 IPV and G2 PCV were highly correlated, whereas they were not significantly correlated in the intervention group. No other pathways in the model were statistically significant.

**Model 2: Intergenerational Transmission of Violence and Its Impact on G3 SDQ Conduct Problems Scores (Figure 3)**

The G3 SDQ Conduct Problems model replicated the G3 Total Difficulties Score model in most ways. After examining chi-squared difference tests, the model fit best when one of the aforementioned eight paths was freed to vary across Fast Track intervention and control groups, and it was once again the path from G1 IPV predicting G2 PCV (*Χ^2^*(1) = 13.77, *p* < .01). This final model is depicted in Figure 3.

This model also supported our first and second study hypotheses in the exact same way as the G3 Total Difficulties score model. Specifically, for G2s in the control group, if G1 parents reported engaging in higher IPV when G2s were 6-14 years old, then those G2s engaged in higher levels of PCV at age 34, which were associated with greater G3 conduct problems. However, the Fast Track intervention protected against this intergenerational transmission of violence because the link between G1 IPV and G2 PCV was only significant in the control group, not the intervention group. This once again made the entire G1 IPV to G2 PCV to G3 conduct problems pathway statistically significant in the control group (Indirect Effect = 0.27, SE = 0.13, 95% CI: 0.01-0.53, *p* = .03) but not the intervention group (Indirect Effect = -0.11, SE = 0.09, 95% CI: -0.29-0.07, *p* = .23).

The other significant findings were also identical; in both treatment and control groups, higher G2 PCV predicted more G3 conduct problems, and G1 IPV and G1 PCV were highly correlated. Once again, G2 IPV and G2 PCV were only highly correlated in the control group. No other model pathways were statistically significant.

**Model 3: Intergenerational Transmission of Violence and Its Impact on G3 SDQ Hyperactivity Scores (Figure 4)**

The G3 SDQ Hyperactivity model replicates the G3 Total Difficulties Score and G3 conduct problems models. As with those aforementioned models, this model fit best when the path from G1 IPV predicting G2 PCV was freed to vary across groups (*Χ^2^*(1) = 12.95, *p* < .01; Figure 4).

This model also supported our first and second study hypotheses in the exact same way as the G3 Total Difficulties and G3 conduct problems models. Specifically, for G2s in the control group, if G1 parents reported engaging in higher IPV when G2s were 6-14 years old, then those G2s engaged in higher levels of PCV at age 34, which were associated with greater G3 difficulties with hyperactivity. However, the Fast Track intervention protected against this intergenerational transmission of violence because the link between G1 IPV and G2 PCV was only significant in the control group, not the intervention group. This once again made the entire G1 IPV to G2 PCV to G3 hyperactivity problems pathway statistically significant in the control group (Indirect Effect = 0.24, SE = 0.12, 95% CI: 0.005 – 0.48, *p* = .05) but not the intervention group (Indirect Effect = -0.09, SE = 0.08, 95% CI: -0.25-0.07, *p* = .25).

The other significant findings were also identical; in both treatment and control group higher G2 violent parenting predicted more G3 hyperactivity problems and G1 IPV and G1 PCV were highly correlated. Once again, G2 IPV and G2 PCV were only highly correlated in the control group. No other model pathways were statistically significant.

**Model 4: Intergenerational Transmission of Violence and Its Impact on G3 SDQ Emotional Problems Scores (Figure 5)**

The G3 SDQ Emotional Problems model differed somewhat from the first two models described above. Similar to Models 1 and 2, the G3 SDQ Emotional Problems model fit best when the path from G1 IPV predicting G2 PCV was freed to vary (*Χ^2^*(1) = 13.08, *p* < .01) across groups (Figure 5).

However, unlike in Models 1 and 2, hypotheses 1 and 2 were only partially supported. Specifically, once again, higher G1 IPV predicted higher G2 PCV in the control group but not the Fast Track treatment group. However, in both groups, G2 PCV was not significantly associated with G3 SDQ emotional problems. Instead, higher G2 IPV was significantly associated with higher G3 SDQ emotional problems in both groups. Therefore, these results partially supported hypothesis 1 because G1 violent behavior (in the form of IPV) did predict G2 violent behavior (in the form of PCV) in the control group, and they partially supported hypothesis 2 because FT participation protected against this link in the treatment group. However, both hypotheses were not fully supported because G2 violent parenting was not associated with G3 emotional problems and consequently the mediating path from G1 IPV to G2 PCV to G3 emotional problems was not statistically significant in either the control (Indirect Effect = 0.11, SE = 0.07, 95% CI: -0.25-0.03, *p* = .11) or intervention (Indirect Effect = -0.04, SE = 0.05, 95% CI: -0.14-0.06, *p* = .40) group.

**Model 5: Intergenerational Transmission of Violence and Its Impact on G3 SDQ Peer Problems Scores (Figure 6)**

The G3 SDQ Peer Problems model results in many ways fell between those of the G3 total difficulties, conduct problems, and hyperactivity models on the one hand and the G3 emotional problems model on the other. As with the G3 total difficulties score model, this model fit best when two paths were freed to vary: (1) the path from G1 IPV predicting G2 PCV (*Χ^2^*(1) = 13.33, *p* < .01) and (2) the path from G1 IPV predicting G3 SDQ Peer Problems scores (*Χ^2^*(1) = 4.26, *p* = .04). This final model is depicted in Figure 6.

Moreover, the significance of the individual paths in this model replicated the G3 total difficulties, G3 conduct problems, and G3 hyperactivity models. Specifically, for G2s in the control group, if G1 parents reported engaging in higher IPV when G2s were 6-14 years old, then those G2s engaged in higher levels of PCV at age 34, which were associated with greater G3 peer problems. However, the Fast Track intervention protected against this intergenerational transmission of violence because the link between G1 IPV and G2 PCV was only significant in the control group, not the intervention group. Where this model differs from the G3 total difficulties, conduct problems, and hyperactivity models is when the entire intergenerational mediating pathway is examined. When this is done, the mediating pathway from G1 IPV to G2 PCV to G3 peer problems is not statistically significant in either the control (Indirect Effect = 0.24, SE = 0.13, 95% CI: -0.02-0.50, *p* = .07) or intervention (Indirect Effect = -0.10, SE = 0.08, 95% CI: -0.26-0.06, *p* = .21) groups. Therefore, like the G3 emotional problems model, hypotheses 1 and 2 are only partially supported in this G3 peer problems model.

The other significant findings were identical to the total difficulties, conduct problems, and hyperactivity models; in both the treatment and control groups, higher G2 PCV predicted more G3 peer problems, and G1 IPV and G1 violent parenting were highly correlated. Once again, G2 IPV and G2 PCV were only highly correlated in the control group. No other model pathways were statistically significant.
